# Supplementary material for: iPSC-derived cerebral organoids reveal mitochondrial, inflammatory and neuronal vulnerabilities in bipolar disorder
Source: Transl Psychiatry. 2025 Aug 25;15:315. doi: 10.1038/s41398-025-03529-7 (PMC12379146; doi:10.1038/s41398-025-03529-7)
Supplement: Supplementary file 1 — Supplementary Table 1 [file 41398_2025_3529_MOESM1_ESM.docx]

# Supplementary Table 1: Clinical Data and Demographics

| Participant number | BD 001 | BD 002 | BD 003 | BD 004 | BD 008 | Participant number.1 | CT 001 | CT 002 | CT 003 | CT 004 | CT 006 |
| --- | --- | --- | --- | --- | --- | --- | --- | --- | --- | --- | --- |
| Interviewer | BA | BA | BA | RZ | RZ | Interviewer | BA | BA | BA | RZ | RZ |
| Date of Assessment | 2018-09-12 00:00:00 | 2018-10-18 00:00:00 | 2019-01-16 00:00:00 | 2019-01-16 00:00:00 | 2019-05-22 00:00:00 | Date of Assessment | 2019-10-04 00:00:00 | 2019-02-15 00:00:00 | 2019-04-11 00:00:00 | 2019-05-23 00:00:00 | 2019-05-28 00:00:00 |
| Gender | F | F | F | F | F | Gender | F | F | F | F | F |
| Age | 38 | 59 | 36 | 42 | 35 | Age | 39 | 42 | 37 | 55 | 31 |
| Height | 1.69 | 1.6 | 1.6 | 1.71 | 1.65 | Height | 1.71 | 1.64 | 1.68 | 1.56 | 1.59 |
| Weight | 85 | 85 | 67 | 105.5 | 65 | Weight | 70 | 61 | 61 | 60 | 60 |
| BMI | 29.76086271489094 | 33.20312499999999 | 26.171874999999996 | 36.07947744605178 |  | BMI | 23.938989774631512 | 22.679952409280194 | 21.612811791383223 | 24.7 | 23.73323840037973 |
| Medical comorbidity - Current | 0 | 1 | 1 | 0 | 1 | Control | 1 | 1 | 1 | 1 | 1 |
| MC Current - Comment | . | Asthma, Osteoarthritis | Complex Regional Pain Syndrome (CRPS) | . | Dyslipidaemia | Medical Comorbidity - Current | 0 | 0 | 0 | 1 | 0 |
| Med Comorbidity - Past | 0 | 0 | 1 | 0 | 0 | MC Current - Comment | . | . | . | Hypothyroidism | . |
| MC - Past - Comment | . | 0 | Stomach bypass in 2011 | 0 | . | Med Comorbidity - Past | 0 | 0 | 0 | 0 | 0 |
| Current Medication (CM): Lithium | 1 | 0 | 0 | 0 | 0 | MC - Past - Comment | . | . | . | . | . |
| CM - Lithium - Dosage | 200mg | . | . | . | . | Current Medication (CM): Lithium | 0 | 0 | 0 | 0 | 0 |
| CM- Lithium Start date | 2016 | . | . | . | . | CM - Lithium - Dosage | . | . | . | . | . |
| Current Medication (CM): Anticonlvulsant | 0 | 0 | 0 | 0 | 1 | CM- Lithium Start date | . | . | . | . | . |
| CM - AC drugs | 0 | . | . | 0 | Lamotrigine 200mg | Current Medication (CM): Anticonlvulsant | 0 | 0 | 0 | 0 | 0 |
| CM AC Start date | 0 | . | . |  | 2012 | CM - AC drugs | . | . | . | . | . |
| Current Medication (CM): Antidepressant | 1 | 1 | 1 | 0 | 0 | CM AC Start date | . | . | . | . | . |
| CM - AD drugs | Sertraline 100mg | Venlafaxine 300mg | Sertraline 100mg |  | . | Current Medication (CM): Antidepressant | 0 | 0 | 0 | 0 | 0 |
| CM AD Start date | 2017 | 1993 | 2014 |  | . | CM - AD drugs | . | . | . | . | . |
| Current Medication (CM): Antipsychotic | 0 | 1 | 1 | 1 | 0 | CM AD  Start date | . | . | . | . | . |
| CM - AP drugs | 0 | Quetiapine 300mg | Aripiprazole 15mg | Quetiapine 200mg | . | Current Medication (CM): Antipsychotic | 0 | 0 | 0 | 0 | 0 |
| CM - AP - Start date | 0 |  | 2014 | 2005 | . | CM - AP drugs | . | . | . | . | . |
| Current Medication (CM): Other | 0 | 1 | 1 | 0 | 0 | CM - AP - Start date | . | . | . | . | . |
| CM - Other | Psychotherapy | Clonazepam 2mg; Pravachol 10mg; Nexium 20mg; Vitamin D; Coloxil with Senna; Symbicort inhaler 4 pumps/day | Norflex 200mg; Dilaudid 16mg; Targin (30+15); Jurnista 8mg; Nexium 40mg. | . | . | Current Medication (CM): Other | 1 | 0 | 1 | 1 | 1 |
| CM - Other - Start date | 2008 |  |  | . | . | CM - Other | Levornogestrel | . | Iron + Vit B12 | Levothyroxine 75mg | Desogestrel + ethinyl estradiol (Mirvala) |
| Lithium Responder | 1 | 0 | 0 | 0 | 0 | CM - Other - Start date | 2017 | . | 2019 | 2017 | 2013 |
| Lithium non-responder | 0 | 1 | 1 | 1 | 1 | Lithium Responder | . | . | . | . | . |
| Tobacco use | 0 | 0 | 1 | 0 | 0 | Lithium non-responder | . | . | . | . | . |
| Cigarettes/day | . | . | 10 to 15 | . | . | Tobacco use | 0 | 0 | 0 | 0 | 0 |
| Cigarettes/ years of use | . | . | 5 | . | . | Cigarettes/day | . | . | . | . | . |
| Alcohol use | 1 | 0 | 1 | 0 | 1 | Cigarettes/ years of use | . | . | . | . | . |
| Drinks/ week | 13 | 0 | 4 | . | 2 | Alcohol use | 1 | 1 | 1 | 1 | 1 |
| Recreational Drugs | 0 | 0 | 0 | 0 | 0 | Drinks/ week | 3 | 6 | 4 | 3 | 9 |
| RD Comment | . | . | . |  | . | Recreational Drugs | 0 | 0 | 0 | 0 | 0 |
| Current Diagnosis:  1 (BD 1);  2 (BD 2);  3 (BD NOS) | 2 | 1 | 1 | 1 | 2 | RD Comment | . | . | . |  | . |
| Other diagnosis | 0 | 0 | 0 | 0 | 0 | Current Diagnosis:  1 (BD 1);  2 (BD 2);  3 (Control) | 3 | 3 | 3 | 3 | 3 |
| Age when sx first noticed | 16 | 29 | 23 | 26 | 21 | Other diagnosis | 0 | 0 | 0 | 0 | 0 |
| Age of definitive diagnosis | 30 | 34 | 24 | 26 | 22 | Age when sx first noticed | . | . | . | . | . |
| Number of psych hospitalizations | 0 | 20 | > 10 | 4 | 1 | Age of definitive diagnosis | . | . | . | . | . |
| Duration of ilness since 1st sx | 22 | 30 | 13 | 15 | 14 | Number of psych hospitalizations | . | . | . | . | . |
| Duration of ilness since diagnosis | 8 | 26 | 12 | 15 | 13 | Duration of ilness since 1st sx | . | . | . | . | . |
| Number of depressive episodes (approx.) | 15 | >30 | > 10 | 8 | 2 | Duration of ilness since diagnosis | . | . | . | . | . |
| Number of manic episodes (approx.) | 15 | 10 to 15 | 2 | 8 |  | Number of depressive episodes (approx.) | . | . | . | . | . |
| Number of suicide attempts | 2 | 0 | 0 | 0 | 1 | Number of manic episodes (approx.) | . | . | . | . | . |
| Family history (FH):  1(yes)/ 0 (no).  For the following questions:  1 (Father), 2 (Mother),  3 (Siblings), 4 (Grandparents) | 1 | 1 | 1 | 1 | 1 | Number of suicide attempts | . | . | . | . | . |
| FH Bipolar: | 0 | 1? | 0 | 6 | 0 | Family history (FH):  1(yes)/ 0 (no). For the following questions:  1 (Father), 2 (Mother),  3 (Siblings), 4 (Grandparents), 5 (Other) | 1 | 0 | 0 | 0 | 0 |
| FH Schizophrenia | 0 | 0 | 0 | 0 | 0 | FH Bipolar: | 0 | 0 | 0 |  | 0 |
| FH Schizoaffective | 0 | 0 | 0 | 0 | 0 | FH Schizophrenia | 0 | 0 | 0 |  | 0 |
| FH Depression | 1,4 | 1,2,3 | 2,3,4 | 2,4,6 | 3 | FH Schizoaffective | 0 | 0 | 0 |  | 0 |
| FH Post Natal Depression | 0 | 0 | 0 | 0 | 0 | FH Depression | 2,3 | 0 | 0 |  | 0 |
| FH Other | Anxiety = 2 | 0 | 1 | 1 | Anxiety (Daughter) | FH Post Natal Depression | 0 | 0 | 0 |  | 0 |
| SCID 5: |  |  |  |  |  | FH Other | 0 | 0 | 0 |  | 0 |
| Marital status | 1 | 5 | 1 | 1 | 1 | SCID 5: |  |  |  |  |  |
| Education | 6 | 4 | 7 | 7 | 7 | Marital status | 5 | 1 | 1 | 1 | 5 |
| Employment status | 2 | 8 | 8 | 7 | 2 | Education | 8 | 8 | 6 | 7 | 8 |
| Suicidal ideation (Past week) | 0 | 0 | 0 | 0 | 0 | Employment status | 1 | 2 | 1 | 1 | 2 |
| MDE - Current | 0 | 1 | 0 | 0 | 0 | MDE - Current | 0 | 0 | 0 | 0 | 0 |
| MDE - Past | 1 | 1 | 1 | 1 | 1 | MDE - Past | 0 | 0 | 0 | 0 | 0 |
| Number of previous MDE | 15 | >30 | >10 | 8 | 2 | Number of previous MDE | 0 | 0 | 0 | 0 | 0 |
| MDE- Appettite pattern | 0 | 0 | 2 | 0 | 0 | MDE- Appettite pattern | . | . | . | . | . |
| MDE Sleep Pattern | 2 | 2 | 2 | 1 | 2 | MDE Sleep Pattern | . | . | . | . | . |
| MDE - Psychotmotor pattern | 2 | 2 | 2 | 0 | 2 | MDE - Psychotmotor pattern | . | . | . | . | . |
| MDE - atypical features | 1 | 1 | 1 | 0 | 0 | MDE - atypical features | . | . | . | . | . |
| Manic Episode - Current | 0 | 0 | 0 | 0 | 0 | Manic Episode - Current | 0 | 0 | 0 | 0 | 0 |
| Manic Episode - Past | 0 | 1 | 1 | 1 | 0 | Manic Episode - Past | 0 | 0 | 0 | 0 | 0 |
| Number of previous manic episodes | 0 | 12 | 1 | 8 | 0 | Number of previous manic episodes | . | . | . | . | . |
| Hypomanic Episode - Current | 0 | 0 | 0 | 0 | 0 | Hypomanic Episode - Current | 0 | 0 | 0 | 0 | 0 |
| Hypomanic Episode - Past | 1 | 0 | 1 | 0 | 0 | Hypomanic Episode - Past | 0 | 0 | 0 | 0 | 0 |
| Nuber of previous Hypomanic episodes | 15 | 0 | 1 | 0 | 0 | Nuber of previous Hypomanic episodes | . | . | . | . | . |
| Premenstrual dysphoric disorder (Past 12 months) | 0 | 0 | 1 | 1 | 0 | Premenstrual dysphoric disorder (Past 12 months) | 0 | 0 | 0 | 0 | 0 |
| Psychotic symptoms - Present | 0 | 0 | 0 | 0 | 0 | Psychotic symptoms - Present | 0 | 0 | 0 | 0 | 0 |
| Psychotic symptoms - Past | 0 | 1 | 0 | 1 | 0 | Psychotic symptoms - Past | 0 | 0 | 0 | 0 | 0 |
| Bipolar 1 Disorder-Without PF | 0 | 0 | 1 | 0 | 0 | Bipolar 1 Disorder-Without PF | 0 | 0 | 0 | 0 | 0 |
| Bipolar 1 Disorder-With PF | 0 | 1 | 0 | 1 | 0 | Bipolar 1 Disorder-With PF | 0 | 0 | 0 | 0 | 0 |
| Bipolar II | 1 | 0 | 0 | 0 | 1 | Bipolar II | 0 | 0 | 0 | 0 | 0 |
| Bipolar NOS | 0 | 0 | 0 | 0 | 0 | Current or most recent episode  (1 = depressive; 2 = hypo/manic) | . | . | . | . | . |
| Current or most recent episode (1 = depressive; 2 = hypo/manic) | 1 | 1 | 1 | 1 | 2 | Months since last mood episode | . | . | . | . | . |
| Months since last mood episode | 24 | 0 | 0 | 6 | >12 | Rapid Cycling | . | . | . | . | . |
| Rapid Cycling | 0 | 0 | 0 | 0 | 1 | Mixed Features | . | . | . | . | . |
| Mixed Features | 0 | 0 | 0 | 0 | 0 | Seasonal Pattern | . | . | . | . | . |
| Seasonal Pattern | 0 | 1 | 0 | 0 | 0 | Suicidal ideation (Past week) | 0 | 0 | 0 | 0 | 0 |
| Panic Disorder, Current Without Agoraphobia | 0 | 0 | 0 | 0 | 0 | Panic Disorder, Current Without Agoraphobia | 0 | 0 | 0 | 0 | 0 |
| Panic Disorder, Current With Agoraphobia | 0 | 1 | 0 | 0 | 0 | Panic Disorder, Current With Agoraphobia | 0 | 0 | 0 | 0 | 0 |
| Agoraphobia, Current Without history of panic dis. | 0 | 0 | 0 | 0 | 0 | Agoraphobia, Current Without history of panic disorder | 0 | 0 | 0 | 0 | 0 |
| Agoraphobia, Current without current panic disorder but with a past history of panic disorder | 0 | 0 | 0 | 0 | 0 | Agorphobia, Current without current panic disorder but with a past history of panic disorder | 0 | 0 | 0 | 0 | 0 |
| Agoraphobia, Current without history of limited symptom attacks | 0 | 0 | 0 | 0 | 0 | Agoraphobia, Current without history of limited symptom attacks | 0 | 0 | 0 | 0 | 0 |
| Past Panic Disorder | 1 | 1 | 0 | 0 | 0 | Past Panic Disorder | 0 | 0 | 0 | 0 | 0 |
| Social Phobia - Current | 0 | 1 | 0 | 0 | 0 | Social Phobia (SAD) - Current | 0 | 0 | 0 | 0 | 0 |
| Social Phobia - Past | 1 | 1 | 0 | 0 | 0 | Social Phobia - Past | 0 | 0 | 0 | 0 | 0 |
| Specific Phobia - Current | 0 | 1 | 1 | 0 | 1 | Specific Phobia - Current | 0 | 0 | 0 | 0 | 0 |
| Specific Phobia - Past | 0 | 1 | 1 | 0 | 1 | Specific phobia - Past | 0 | 0 | 0 | 0 | 0 |
| Generalized Anxiety Disorder (GAD) - Current | 0 | 1 | 0 | 0 | 0 | Generalized Anxiety Disorder (GAD) - Current | 0 | 0 | 0 | 0 | 0 |
| GAD - Past | 0 | 1 | 1 | 0 | 0 | GAD - Past | 0 | 0 | 0 | 0 | 0 |
| ACE - 1 | 2 | 1 | 1 | 1 | 2 | ACE - 1 | 2 | 2 | 2 | 2 | 2 |
| ACE - 2 | 2 | 2 | 2 | 2 | 2 | ACE - 2 | 2 | 2 | 2 | 2 | 1 |
| ACE -3 | 2 | 2 | 1 | 2 | 2 | ACE -3 | 2 | 2 | 2 | 2 | 2 |
| ACE - 4 | 2 | 2 | 2 | 2 | 2 | ACE - 4 | 2 | 2 | 2 | 2 | 2 |
| ACE - 5 | 2 | 2 | 2 | 2 | 2 | ACE - 5 | 2 | 2 | 2 | 2 | 1 |
| ACE - 6 | 1 | 1 | 1 | 1 | 1 | ACE - 6 | 1 | 1 | 1 | 3 | 2 |
| ACE-7 | 1 | 1 | 1 | 1 | 1 | ACE-7 | 1 | 1 | 1 | 1 | 1 |
| ACE - 8 | 1 | 1 | 1 | 2 | 1 | ACE - 8 | 2 | 1 | 1 | 1 | 1 |
| ACE - 9 | 1 | 1 | 1 | 1 | 1 | ACE - 9 | 1 | 1 | 2 | 1 | 1 |
| ACE - 10 | 1 | 1 | 1 | 1 | 1 | ACE - 10 | 1 | 1 | 1 | 1 | 1 |
| ACE - 11 | 1 | 1 | 1 | 1 | 1 | ACE - 11 | 1 | 1 | 1 | 1 | 1 |
|  |  |  |  |  |  | ACE -Total |  |  |  |  |  |

Supplementary Table 1. This table summarizes clinical and demographic data as well as family history of psychiatric disorders.
