# Supplementary material for: iPSC-derived cerebral organoids reveal mitochondrial, inflammatory and neuronal vulnerabilities in bipolar disorder
Source: Transl Psychiatry. 2025 Aug 25;15:315. doi: 10.1038/s41398-025-03529-7 (PMC12379146; doi:10.1038/s41398-025-03529-7)
Supplement: Supplementary file 2 — Supplementary Table 2 [file 41398_2025_3529_MOESM2_ESM.docx]

Supplementary Table 2. Summary of Karyotyping Results

| Participant # | Karyotyping Result | Participant # | Karyotyping Result |
| --- | --- | --- | --- |
| CT001 | Mosaic, 46,X, - X, +mar/46,XX[20] | BD004 | Abnormal, 46XX, add(11)(q23)[cp6] |
| CT006 | Mosaic, 46-48, XX, add(9)(q?13)[cp2]/46,XX[16] | B008 | Mosaic, 46, XX, t(2;11)(q2?3;q23)[2]/46,XX[5] |

**Supplementary Table 2.** Summary karyotype analysis of iPSC lines. Lines which exhibited an abnormal karyotype, thus were not include in the study.
