# Supplementary material for: iPSC-derived cerebral organoids reveal mitochondrial, inflammatory and neuronal vulnerabilities in bipolar disorder
Source: Transl Psychiatry. 2025 Aug 25;15:315. doi: 10.1038/s41398-025-03529-7 (PMC12379146; doi:10.1038/s41398-025-03529-7)
Supplement: Supplementary file 3 — Supplementary Figures [file 41398_2025_3529_MOESM3_ESM.pptx]

## Slide 1
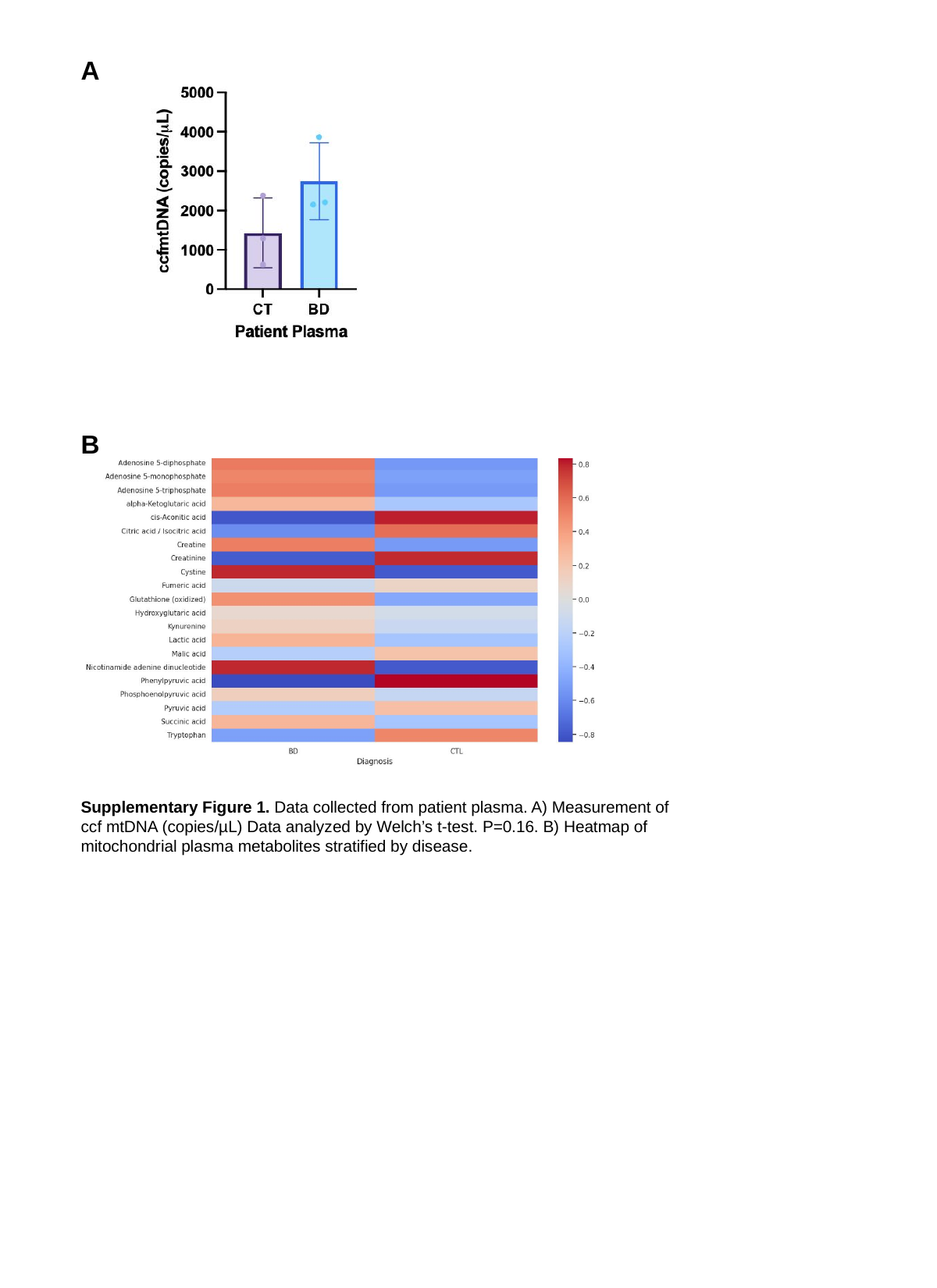

A
B
Supplementary Figure 1. Data collected from patient plasma. A) Measurement of ccf mtDNA (copies/µL) Data analyzed by Welch’s t-test. P=0.16. B) Heatmap of mitochondrial plasma metabolites stratified by disease.

## Slide 2
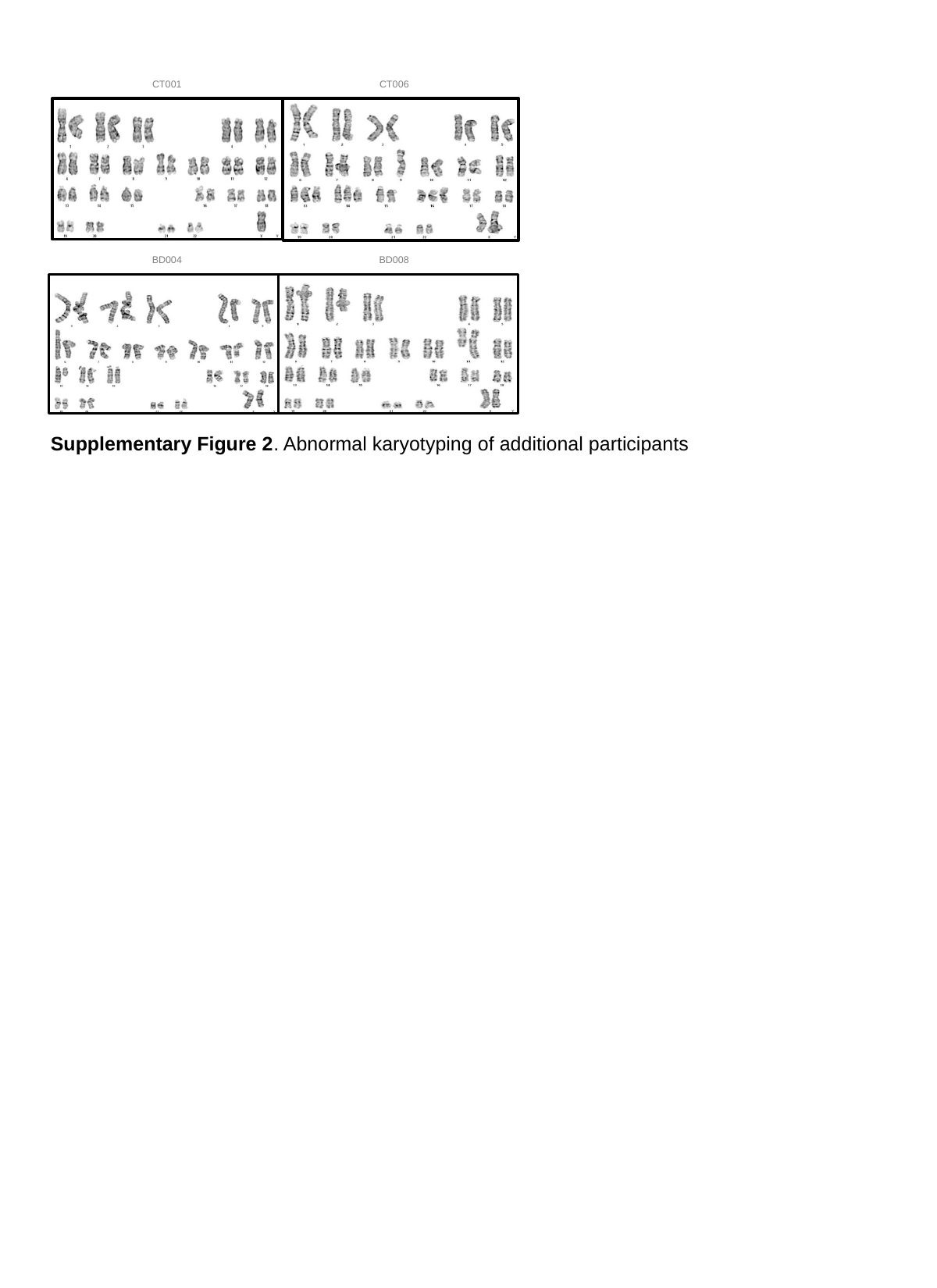

CT001
CT006
BD004
BD008
Supplementary Figure 2. Abnormal karyotyping of additional participants

## Slide 3
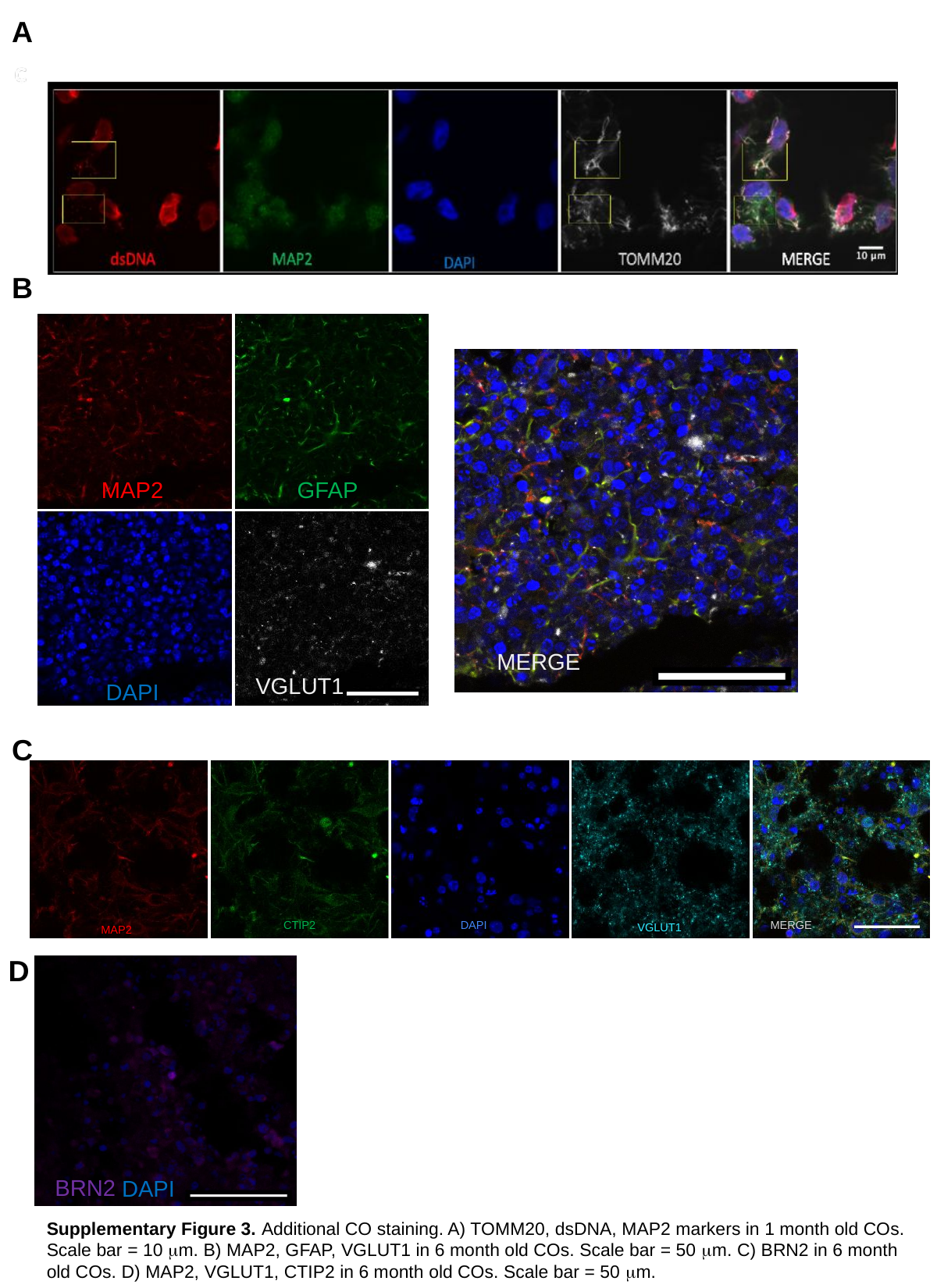

A
B
MAP2
GFAP
MERGE
VGLUT1
DAPI
C
MERGE
CTIP2
DAPI
VGLUT1
MAP2
D
BRN2
DAPI
Supplementary Figure 3. Additional CO staining. A) TOMM20, dsDNA, MAP2 markers in 1 month old COs. Scale bar = 10 μm. B) MAP2, GFAP, VGLUT1 in 6 month old COs. Scale bar = 50 μm. C) BRN2 in 6 month old COs. D) MAP2, VGLUT1, CTIP2 in 6 month old COs. Scale bar = 50 μm.
